# Case report: Application of nirmatrelvir/ritonavir to treat COVID-19 in a severe aplastic anemia child after allogeneic hematopoietic stem cell transplantation

**DOI:** 10.3389/fped.2022.935118

**Published:** 2022-08-08

**Authors:** Jinhua Huang, Di Yin, Xia Qin, Mei Yu, Beilei Jiang, Jing Chen, Qing Cao, Zhiguo Tang

**Affiliations:** ^1^Hefei First People’s Hospital, Hefei, China; ^2^Department of Hematology and Oncology, Shanghai Children’s Medical Center, Shanghai Jiao Tong University School of Medicine, Shanghai, China; ^3^Department of Infectious Disease, Shanghai Children’s Medical Center, Shanghai Jiao Tong University School of Medicine, Shanghai, China

**Keywords:** COVID-19, children, Paxlovid, severe aplastic anemia, transplantation, Omicron

## Abstract

We present a case report of successful treatment with nirmatrelvir/ritonavir (Paxlvoid) for a severe aplastic anemia child with COVID-19, cytopenia, and mixed chimerism of donor hematopoietic cells at 3 months after allogeneic hematopoietic stem cell transplantation. After the 5-day entire course of treatment, the clinical symptoms were relieved, cycle threshold values of ORF1a/b and N genes increased from 22.60 and 22.15 to 34.52 and 33.84, respectively, and the peripheral blood counts gradually recovered without graft failure. Nirmatrelvir/ritonavir can effectively inhibit the replication of SARS-CoV-2 without any significant adverse effects.

## Introduction

Coronavirus disease 2019 (COVID-19) has emerged as a global pandemic. The SARS-CoV-2 Omicron variant outbreak has turned out to be a massive burden on the healthcare system all over the world. Notably, the number of confirmed Omicron cases has rapidly increased around China from early 2022. The proportion of severe COVID-19 caused by all variant of SARS-CoV-2 in children is 7%, lower than in adults (25.6%) (1). Children’s most common clinical manifestations are fever (51%) and cough (41%) (2), however, there is currently little experience treating COVID-19 in children, especially with underlying illnesses. Aplastic anemia (AA) is a bone marrow failure syndrome characterized by peripheral blood pancytopenia and empty bone marrow (3). Allogeneic hematopoietic stem cell transplantation (allo-HSCT) from a HLA-identical family donor is the first-line therapy in children (4). Here, we report the case of using nirmatrelvir/ritonavir (Paxlovid) to treat COVID-19 in a severe AA child after allo-HSCT.

## Case description

A 5-year-old boy was admitted to Shanghai children’s medical center with pancytopenia. Bone marrow aspiration and biopsy showed markedly hypocellular marrow without abnormal cells. Excluding other causes of pancytopenia and inherited bone marrow failure disorders, acquired severe AA was diagnosed. He received a peripheral blood stem cell transplantation from his HLA-identical sister (total nucleated cells 22.6 × 10^8^/kg and CD34^+^ cells 9.2 × 10^6^/kg) on December 14, 2021. The conditioning regimens consisted of fludarabine (200 mg/m^2^), cyclophosphamide (200 mg/kg), and rabbit anti-thymocyte globulin (Thymoglonuline, 9 mg/kg). Due to the positive anti-HLA panel reactive antibodies, a single dose of rituximab (375 mg/m^2^) was given on day −7 to ensure engraftment. Cyclosporine A and methotrexate (15 mg/m^2^ on day +1, 10 mg/m^2^ on day +3 and +6) were used for graft-versus-host disease (GVHD) prophylaxis. Neutrophil and platelet engraftment was observed on day +15 and day +14 with 100% donor chimerism. However, on day +27, the leukocyte and neutrophil count suddenly dropped to 1.4 × 10^9^/L and 0.5 × 10^9^/L. Donor lymphocyte infusion (DLI) was given with a CD3^+^ T lymphocyte dose of 5.8 × 10^6^/kg to prevent graft rejection immediately. After 2 weeks, the leukocyte and neutrophil count increased to 2.0–2.5 × 10^9^/L and 1.0–1.5 × 10^9^/L. Nevertheless, the donor chimerism of whole blood cells and CD3^+^ T lymphocytes decreased to 94.13 and 83.56%, respectively. The second DLI (CD3^+^ T lymphocyte dose 1.7 × 10^8^/kg) was performed on day +42. Three weeks later, mixed chimerism of whole blood cells (93.89%) and CD3^+^ T lymphocytes (87.05%) were still observed. Thus, the third DLI (CD3^+^ T lymphocyte dose 2.9 × 10^8^/kg) was given on day +70. Due to the epidemic of Omicron variant in Shanghai in March 2022, donor chimerism analysis was not performed after the third DLI. There was no evidence of GVHD or other transplant-related complications.

**FIGURE 1:**
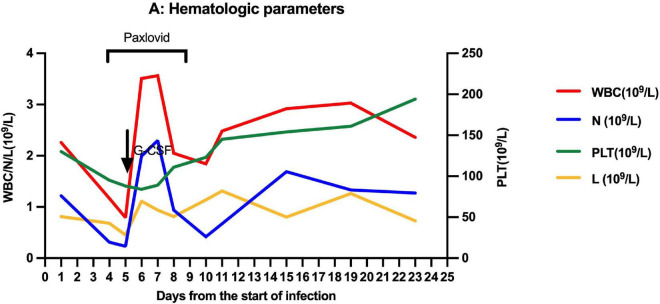
Trend of hematologic parameters over times.

**FIGURE 2:**
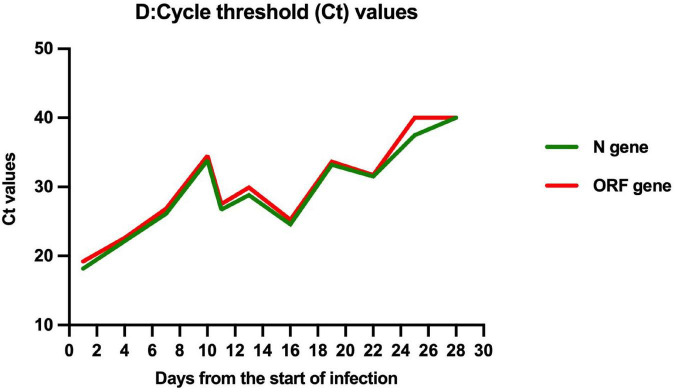
Trend of Ct values of ORF1a/b and N genes over times.

On March 27, 2022, 103 days after allo-HSCT, this boy was referred to our hospital with a 2-day history of low-grade fever, nasal congestion, fatigue, and abdominal pain. The SARS-CoV-2 nucleic acid of the pharyngeal swab was positive. Vital signs were normal, and oxygen saturation was 100% in room air. Routine blood examination revealed the following: white blood cells (WBC) 2.26 × 10^9^/L with 53.6% neutrophils (N), hemoglobin (Hb) 103 g/L, and platelets (PLT) 130 × 10^9^/L. Chest computed tomography showed normal. Therefore, the boy was diagnosed with mild COVID-19. On day 4 (the first day for SARS-CoV-2 positive test designated as day 0), he developed neutropenia, lymphocytopenia, and thrombocytopenia (WBC 1.17 × 10^9^/L, N 0.31 × 10^9^/L, L 0.68 × 10^9^/L, Hb 103 g/L, and PLT 98 × 10^9^/L). A 5-day-course treatment with 150 mg of nirmatrelvir plus 50 mg of ritonavir twice daily began on day 4 in an off-label manner according to Phase 2/3 EPIC-PEDS study after taking consent from the legal guardians and the approval from ethical committee. On day 5, WBC and N counts continued to decrease, and a single dose of granulocyte-colony stimulating factor (G-CSF) was given. However, the effect of G-CSF lasted only two days. The WBC and N counts decreased again on day 8 and stabilized after the complete course of nirmatrelvir/ritonavir treatment (Figure 1), accompanied by the relief of clinical symptoms. The cycle threshold (Ct) values of ORF1a/b and N gene fragments increased from 22.60 and 22.15 to 34.52 and 33.84, respectively, following the 5-day full course of nirmatrelvir/ritonavir treatment (Figure 2). There was no other adverse effect except a mild increase of serum creatinine (73.3 umol/L) and urea nitrogen (12.36 mmol/L) on the post-treatment day 2, which returned to normal on the post-treatment day 5. The donor chimerism and lymphocyte subsets analyses were not performed at the beginning of the SARS-CoV-2 infection. The donor chimerism of whole blood cells was 95.94% on day 9, suggesting stable mixed chimerism without graft failure. According to flow cytometry results, 140/ul CD4^+^T cells (12.32%), 540/ul CD8^+^T cells (47.05%), 110/ul CD19^+^B cells (9.96%), and 200/ul CD16^+^CD56^+^NK cells (17.39%) were detected on day 10, respectively (Table 1). A reduced CD4^+^/CD8^+^ ratio was also observed (Table 1). The SARS-CoV-2 nucleic acid was negative on day 25.

**TABLE 1:** Lymphocyte subsets after the SARS-CoV-2 infection.

	d10	d11	d12	d19	d26
CD3^+^T (cell/ul)	780	890	460	910	660
CD4^+^T (cell/ul)	140	120	90	170	200
CD8^+^T (cell/ul)	540	590	280	620	360
CD19^+^B (cell/ul)	110	30	130	100	120
CD16^+^CD56^+^NK (cell/ul)	200	220	110	250	150
CD3^+^T (%)	67.75	67.38	62.62	72.34	61.31
CD4^+^T (%)	12.32	9.34	12.41	13.58	26.45
CD8^+^T (%)	47.05	44.85	37.87	49.24	31.03
CD19^+^B (%)	9.96	1.99	16.98	8.27	19.59
CD16^+^CD56^+^NK (%)	17.39	17.06	14.91	19.64	19.08
CD4^+^/CD8^+^	0.26	0.21	0.33	0.28	0.85

d, days after the SARS-CoV-19 infection.

## Discussion

The pathogenesis of COVID-19 is characterized by an initial viral phase followed by a inflammatory phase (5). This hyper-inflammatory state is the cause of high mortality rate in patients who develop it (5). Therefore, the treatment of SARS-CoV-2 infection is based on the initial control of the virus and/or subsequently, in patients who need it, on the control of the hyper-inflammatory response. Immunocompromised persons are at risk of a prolonged viral phase compared to typical 5–10 days reported in the general population; the control of the virus becomes presumably very relevant in these population (6). Nirmatrelvir is an oral antiviral agent which inhibits a SARS-CoV-2 protein to stop the virus from replicating, and ritonavir slows down nirmatrelvir’s breakdown to increase drug concentrations and delay clearance (7). Clinic data from a phase 2/3, double-blind, randomized, controlled trial (EPIC-HR) showed that treatment with nirmatrelvir/ritonavir early in COVID-19 illness could decrease progression to severe disease and quickly reduce SARS-CoV-2 viral load, without evident safety concerns (8). In December 2021, nirmatrelvir/ritonavir was authorized for emergency use by US Food and Drug

Administration under an Emergency Use Authorization for the treatment of mild to moderate COVID-19 in adults and pediatric patients (12 years of age and older weighing at least 40 kg), and who are at high-risk of progression to severe COVID-19, including hospitalization or death (9). Moreover, a phase 2/3, open-label, multi-center, single-arm trial (EPIC-PEDS) has been initiated to evaluate the safety and efficacy of nirmatrelvir/ritonavir in pediatric patients.

Multi-center studies revealed that neutropenia and immunodeficiency were significantly associated with increased disease severity of COVID-19 for the patient after allo-HSCT (10, 11). There has also been reported that SARS-CoV-2 could lead to profound cytopenia and even graft failure in patients after allo-HSCT (12, 13). In our case, this is a 5-year-old boy weighing 17.5 kg diagnosed with mild COVID-19. The reasons why we used nirmatrelvir/ritonavir off-label were as follows: firstly, the boy was 3 months post-transplant and immunosuppressive therapy with cyclosporine A was indispensable; secondly, due to the use of rituximab in the conditioning regimen, the reconstitution of B cells after allo-HSCT was delayed, failing to produce sufficient neutralizing antibodies against SARS-CoV-2; thirdly, the boy experienced mixed chimerism before SARS-CoV-2 infection, and developed neutropenia, lymphocytopenia, and thrombocytopenia after SARS-CoV-2 infection; at that time, donor chimerism analysis was not available and graft failure could not be excluded. Considering many high-risk factors for progression to severe COVID-19, nirmatrelvir/ritonavir was administered according to the dose of EPIC-PEDS trial. To the best of our knowledge, this is the first case report of the use of nirmatrelvir/ritonavir to treat a COVID-19 child with immunodeficiency. After the 5-day full course of nirmatrelvir/ritonavir treatment, the clinical symptoms were obviously relieved, the Ct values were significantly increased, and the peripheral blood counts gradually recovered without graft failure. There was no adverse effect except a transient and mild increase in serum creatinine and urea nitrogen. However, we also observed a decrease in Ct values of ORF1a/b and N genes after 5-day treatment of nirmatrelvir/ritonavir. It is worth a further study to investigate whether the treatment course should be prolonged for patients with immunodeficiency.

Retrospective analysis from the United Kingdom and Spanish showed that the median time to SARS-CoV-2 clearance was 20–27 days in pediatric patients after allo-HSCT (12, 14). In patients with immunodeficiency, especially those with intensive immunosuppression for GVHD or profound B cell defect, such as previous exposure to rituximab, shedding infectious viral could even last for months (14–16). In our case, the boy was previously exposed to rituximab and still required immunosuppressive therapy with cyclosporine A to prevent GVHD. After the 5-day course of nirmatrelvir/ritonavir treatment, the Ct values of ORF1a/b and N genes increased from 22.60 and 22.15 to 34.52 and 33.84, suggesting that nirmatrelvir/ritonavir can effectively inhibit viral replication *in vivo*. Though the viral clearance time in our case was 25 days, similar to other reports (12, 14), the viral load in the body was reduced, which could be reflected by the Ct values, thus reducing the risk of progression to severe disease (17).

## Conclusion

In summary, we present the first case report of the successful treatment with nirmatrelvir/ritonavir for a severe AA child with COVID-19, cytopenia, and mixed chimerism of donor hematopoietic cells at 3 months after allo-HSCT. Nirmatrelvir/ritonavir can effectively inhibit the replication of SARS-CoV-2 without any significant adverse effects. Nirmatrelvir/ritonavir could be a potential therapy choice for children with COVID-19 and underlying diseases.

## Data availability statement

The original contributions presented in this study are included in the article/supplementary material, further inquiries can be directed to the corresponding authors.

## Ethics statement

The studies involving human participants were reviewed and approved by the Ethics Committee of Hefei First People’s Hospital. Written informed consent to participate in this study was provided by the participants or their legal guardian/next of kin. Written informed consent was obtained from the minor(s)’ legal guardian/next of kin for the publication of any potentially identifiable images or data included in this article.

## Author contributions

JH, DY, and XQ wrote all drafts. MY collected all the references and clinical data. BJ carried out PCR tests. ZT offered conception. QC and JC polished up and finalized the draft. All authors read and approved the final manuscript.
